# Chitin-rich heteroglycan from *Sporothrix schenckii* sensu stricto potentiates fungal clearance in a mouse model of sporotrichosis and promotes macrophages phagocytosis

**DOI:** 10.1186/s12866-021-02243-w

**Published:** 2021-06-25

**Authors:** Lilin Huang, Jing Zhang, Weian Du, Zixian Liang, Meirong Li, Rong Wu, Sanmei Chen, Xuchu Hu, Huaiqiu Huang

**Affiliations:** 1 Department of Dermatology and Venereology, The Third Affiliated Hospital of Sun Yat-Sen University, 600# Tianhe Road, Guangzhou, 510630 China; 2 Department of Pediatrics, Guangdong Women and Children Hospital, Guangzhou, China; 3 Department of Dermatology and Venereology, Sun Yat-sen Memorial Hospital, Sun Yat-sen University, Guangzhou, China; 4 Department of Parasitology, Zhongshan School of Medicine, Sun Yat-sen University, Guangzhou, China

**Keywords:** *Sporothrix schenckii* sensu stricto, Chitin-rich heteroglycan, Immunomodulation, Phagocytosis, Cytokines

## Abstract

**Background:**

Fungal cell wall polysaccharides maintain the integrity of fungi and interact with host immune cells. The immunomodulation of fungal polysaccharides has been demonstrated in previous studies. However, the effect of chitin-rich heteroglycan extracted from *Sporothrix schenckii* *sensu stricto* on the immune response has not been investigated.

**Results:**

In this study, chitin-rich heteroglycan was extracted from *S. schenckii* *sensu stricto*, and immunomodulation was investigated via histopathological analysis of skin lesions in a mouse model of sporotrichosis and evaluation of the phagocytic function and cytokine secretion of macrophages in vitro. The results showed that the skin lesions regressed and granulomatous inflammation was reduced in infected mice within 5 weeks. Moreover, heteroglycan promoted the fungal phagocytosis by macrophages and modulated the cytokine secretion. Heteroglycan upregulated TNF-α expression early at 24 h and IL-12 expression late at 72 h after incubation, which might result from moderate activation of macrophages and contribute to the subsequent adaptive immune response.

**Conclusions:**

Chitin-rich heteroglycan extracted from *S. schenckii* *sensu stricto* potentiated fungal clearance in a mouse model of sporotrichosis. Moreover, chitin-rich heteroglycan promoted fungus phagocytosis by macrophages and modulated cytokines secretion. These results might indicate that chitin-rich heteroglycan could be considered as an immunomodulator used in the treatment of sporotrichosis.

## Introduction

Sporotrichosis is a subcutaneous mycosis caused by traumatic inoculation of the dimorphic fungus *Sporothrix schenckii* complex, including *S. schenckii s str*, *S. globosa*, *S. brasiliensis*, and *S. luriei* [1]. *S. schenckii s str*, which is the most common fungus in this complex, manifests as mycelial form in the soil and plant debris and yeast form in infected animals [2]. The fungal cell wall is composed of many polysaccharides and glycoproteins that show dynamic change due to the impact of culture media and growth conditions [3]. These polysaccharides might be exposed to the surface of the fungal cell wall or released into the circulation during infection and thus have the chance to interact with host immune cells. An increasing number of studies have reported the immunomodulation of chitin and glucan from *Candida albicans* and *Aspergillus fumigatus* which produced inconsistent results [4–7]. Chitin, glucan, and mannan have also been used as nontoxic immune or vaccine adjuvants to enhance the immune response to several antigens in recent years [8]. Chitin and glucans are covered by mannan and glycoprotein on the cell wall of *S. schenckii s str* [9]. Heat killing and drug treatment of fungi were used to investigate the immune activity of polysaccharides from the fungal cell wall in previous studies [3, 10, 11]. We previously found that chitin exposure on the cell wall of *S. schenckii s str* upon curcumin treatment favored the antifungal response in infected mice [12]. Individual purified polysaccharides are extremely difficult to isolate since the fungal cell wall is present in the form of a chitin-glucan complex. Until now, few studies have investigated the immune activity of purified polysaccharides from *S. schenckii s str*. The alkali-insoluble glucan complex from *S. schenckii s str* could increase nitric oxide (NO) production by peritoneal macrophages alone and stimulate secretion of the proinflammatory cytokines IL-1β, IL-18 and IL-17 when cocultured with splenocytes and peritoneal macrophages [13, 14]. There are many polysaccharides on the cell wall of *S. schenckii* complex, and their distribution results in different virulence levels in the *Galleria mellonella* model [15]. This finding also indicates that these polysaccharides might play important roles in modulating the immune response to the *S. schenckii* complex. The aims of this study were to explore the immune activity of heteroglycan in the pathogenesis of sporotrichosis. We observed that chitin-rich heteroglycan exerted an antifungal response in a mouse model of sporotrichosis and modulated fungal phagocytosis and cytokines secretion by macrophages. The results here demonstrated that chitin-rich heteroglycan from *S. schenckii s str* might act as an immunomodulator in a mouse model of sporotrichosis.

## Results

### The components and size of the heteroglycan extracted from *S. schenckii s str*

**Fig. 1:**
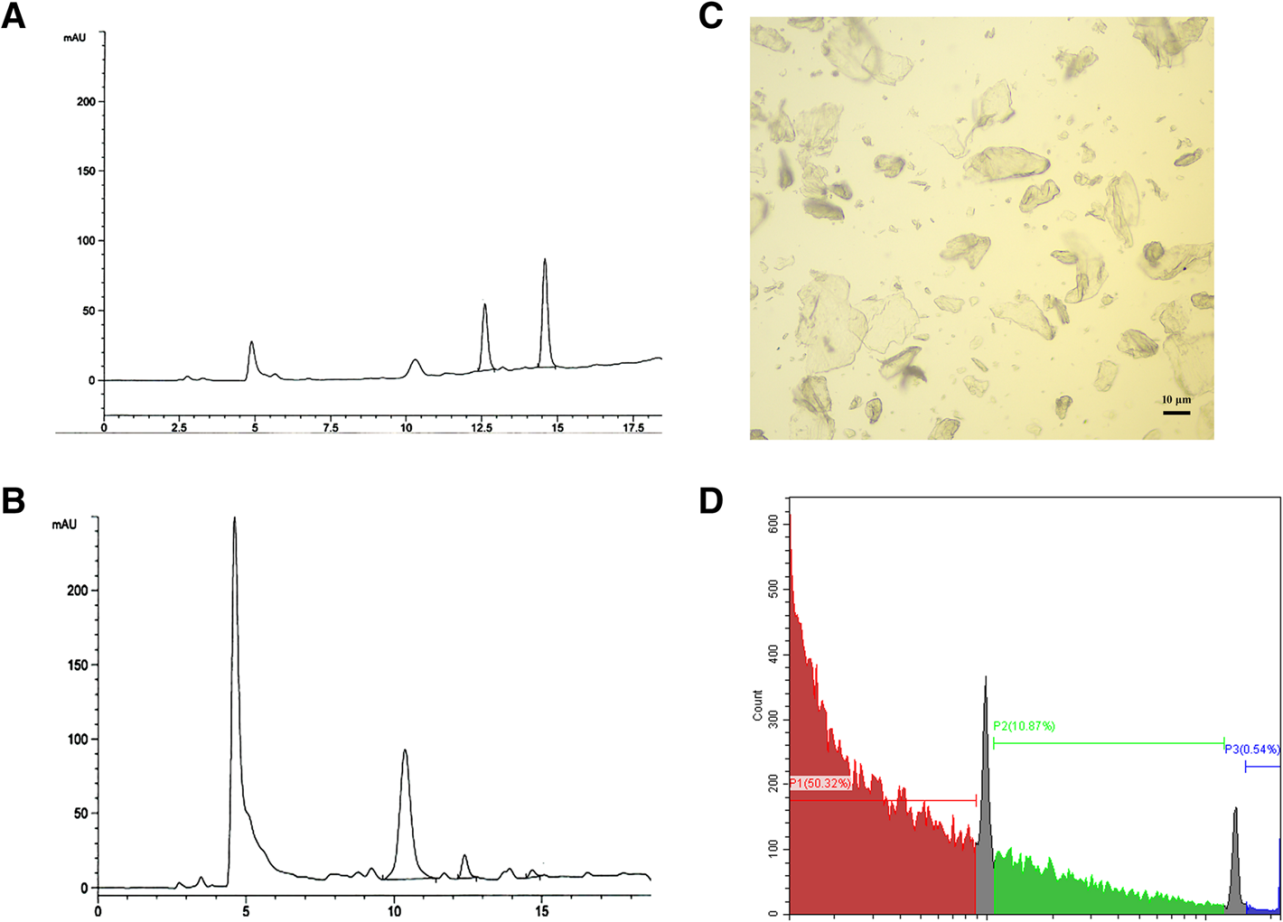
The components and size of the heteroglycan extracted from *S. schenckii s str*. **A** The HPLC chromatogram of carbohydrate standards (GlcN, glucosamine; Man, mannose; Glc, glucose). **B** The components of heteroglycan analyzed by HPLC included chitin (89%), mannan (8.5%), and glucan (2.5%). **C** The microscopic image of heteroglycan microparticles. **D** Heteroglycan size was determined by flow cytometry, which showed that most heteroglycan were less than 10 μm and half of them were less than 2 μm

We extracted heteroglycan from the mycelial form of *S. schenckii s str* and then detected its components by high-performance liquid chromatography (HPLC). The heteroglycan samples were white microparticles in various sizes by microscope (Fig. 1C). The results of HPLC showed that the heteroglycan microparticles were composed of chitin (89%), mannose (8.5%), and glucosamine (2.5%) (Fig. 1B). According to the flow cytometry results, most heteroglycan microparticles were less than 10 μm, and half of them were less than 2 μm in size (Fig. 1D). The heteroglycan microparticles were protein- and lipopolysaccharide-free, and was also negative for microbiological culture.

### Animal model of sporotrichosis

**Fig. 2:**
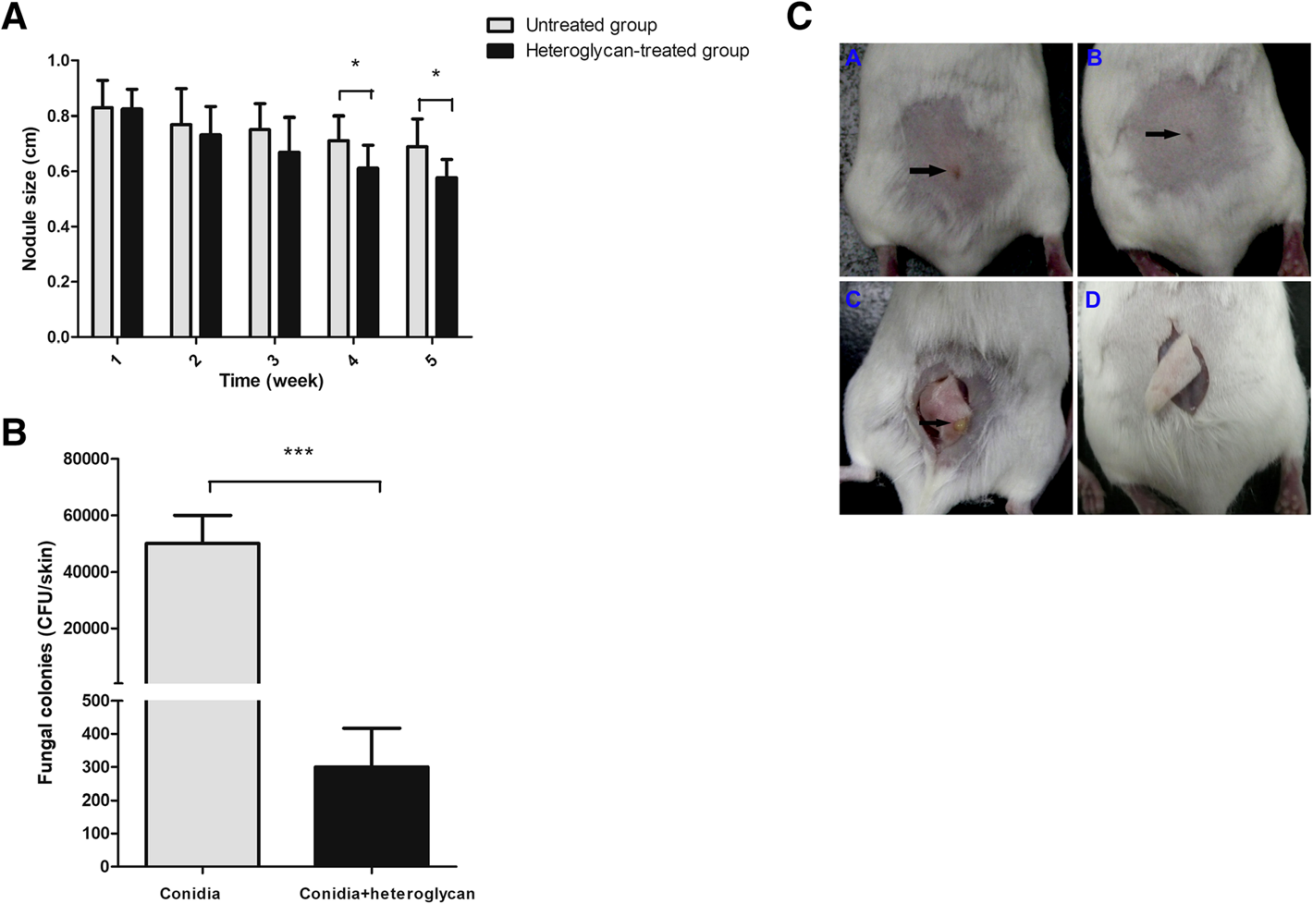
The nodule sizes were monitored weekly in infected mice for 5 weeks (*n* = 20). (a) The nodule sizes of the infected mice with heteroglycan treatment (*n* = 10) decreased significantly compared to those of untreated mice (n = 10) at the end of the 4th and 5th weeks. *, *p* < 0.05. (b) Lesions in the untreated group (A, black arrow) were worse compared to those in the heteroglycan-treated group (B, black arrow) at the end of the 5th week. Yellow pus was extruded from the lesions of the untreated group (C, black arrow), whereas no pus extrusion was observed in the heteroglycan-treated group (D) at the end of the 5th week. (c) There were more fungal colonies from lesions in the untreated group than in the heteroglycan-treated group at the end of the 5th week. ***, *p* < 0.001

Mice were infected with conidia of *S. schenckii s str* to investigate the immune activity of heteroglycan in vivo. Since the proportion of chitin accounted for 89%, we chose 100 μg as the treatment dose of heteroglycan according to a previous study [4]. All the mice in the three groups survived after 5 weeks. We monitored the lesions of conidia-infected mice weekly; they became nodules and reached their maximum size in the 1st week. The nodule sizes did not show significant differences within 3 weeks with or without heteroglycan treatment. However, the lesions of the infected mice with heteroglycan treatment (0.61 ± 0.11 cm, 0.58 ± 0.10 cm*,* respectively) regressed significantly compared to those of the untreated mice (0.71 ± 0.09 cm, 0.69 ± 0.11 cm*,* respectively) at the end of the 4th and 5th weeks (*p* = 0.035, *p* = 0.011*,* respectively) (Fig. 2a). Additionally, yellow pus was extruded from the lesions of the untreated mice (Fig. 2b). Nodules did not develop in the PBS blank group.

### Fungal culture of skin lesions

Local skins with lesions were harvested from conidia-infected mice at the end of the 5th week and homogenized. The homogenates were then grown in SDA at 25 °C for 5 days to determine fungal colonies. More fungal colonies were found in the lesions of the untreated group (5 × 10^4^ ± 1 × 10^4^ CFU/skin) than in the heteroglycan-treated group (300 ± 117 CFU/skin) (*p* < 0.001) (Fig. 2c). No fungal colonies from lung, liver, spleen, or kidney samples were found in the three groups.

### Histopathological analysis of skin lesions

**Fig. 3:**
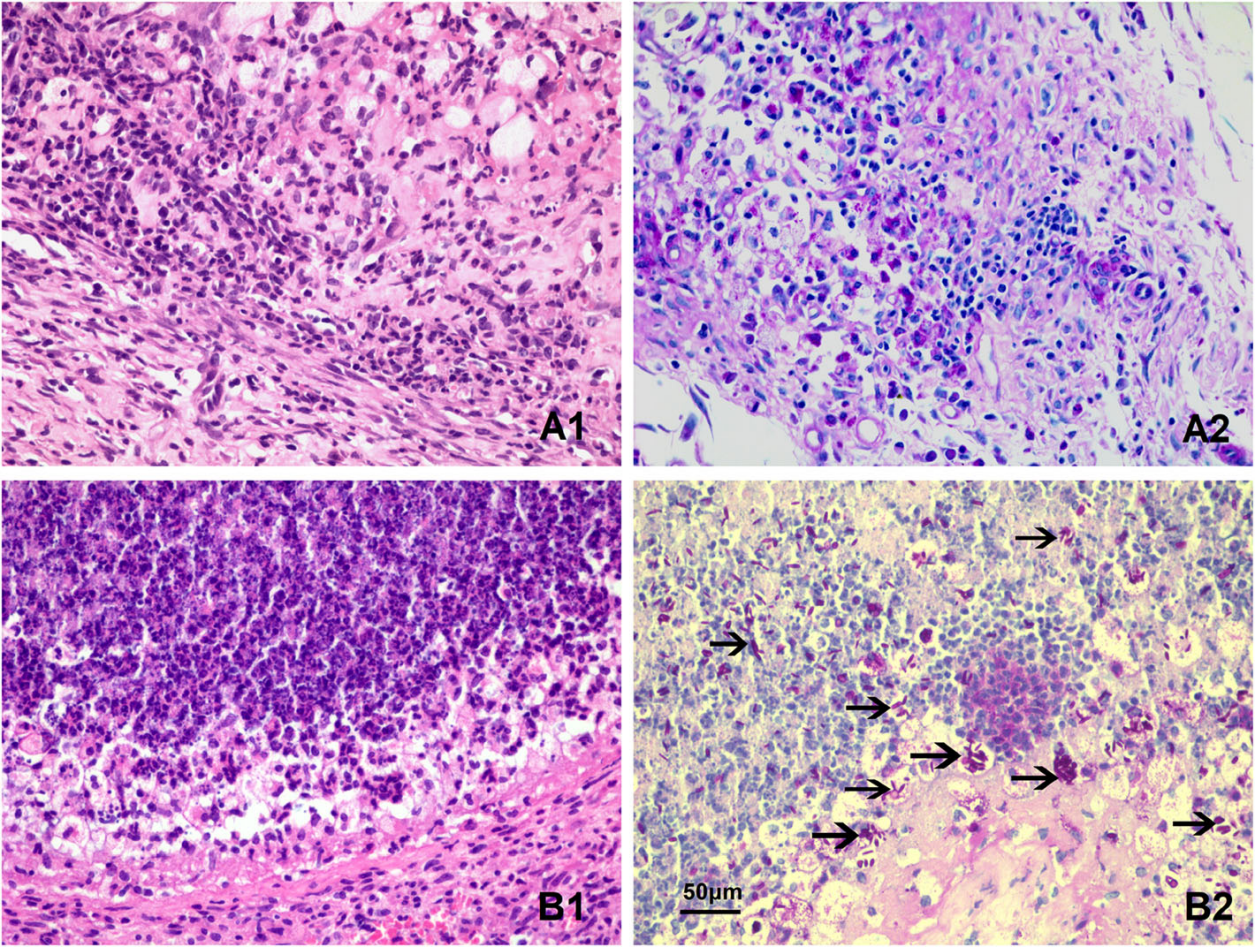
Histopathologic analysis by H&E and PAS staining in infected mice after 5 weeks. A represents the heteroglycan-treated group (n = 10). A1 exhibited reduced granulomatous inflammation with a few mononuclear cells, epithelioid cells, and lymphocyte infiltration in the dermal tissue, as assessed by H&E staining; A2 showed that fungi were rarely observed in the lesion, as assessed by PAS staining. B represents the untreated group (n = 10). B1 exhibited suppurative granulomatous inflammation with numerous neutrophils, mononuclear cells, and lymphocytes in the outer layer of the lesions, as assessed by H&E staining; B2 showed that round, oval and cigar-shaped yeast forms of *S. schenckii s str* (black arrow) were observed in the lesion, as assessed by PAS staining. Scale bar, 50 μm

Lesions from infected mice were assessed by histopathological analysis with hematoxylin and eosin (H&E) and periodic acid-Schiff (PAS) staining. The lesions of the heteroglycan-treated group displayed retracted granulomatous inflammation with reduced immune cell infiltration. Fungus was rarely observed in the nodules of the heteroglycan-treated group following PAS staining (Fig. 3A). In contrast, the lesions of the untreated group exhibited suppurative granulomatous inflammation, which was surrounded by neutrophils, mononuclear cells, and lymphocytes in the outer layer. Many oval- and cigar-shaped yeast forms of *S. schenckii s str* were observed in the nodules of the untreated group following PAS staining (Fig. 3B).

### Phagocytosis assay

**Fig. 4:**
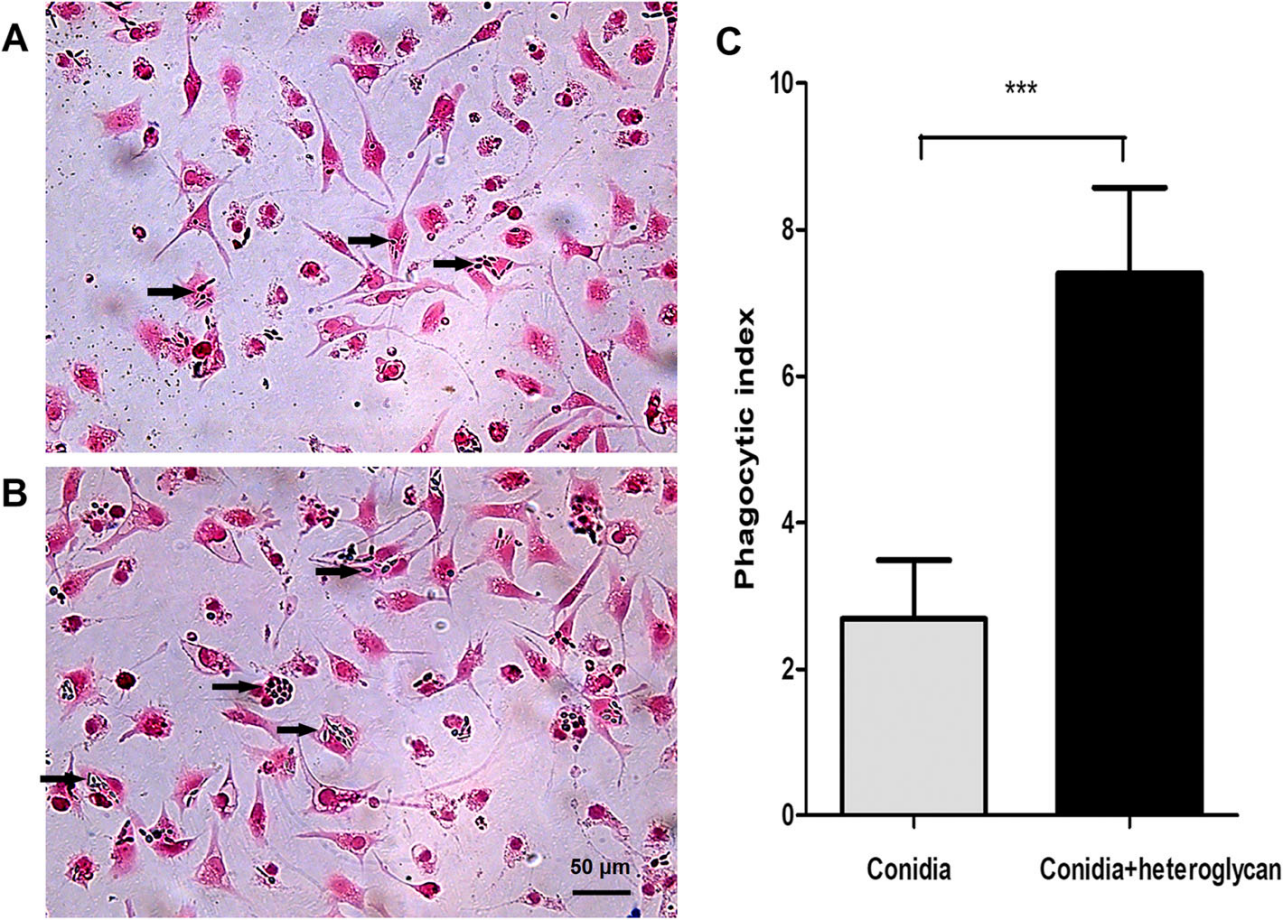
Conidial phagocytosis by macrophages from uninfected mice after incubation for 48 h. Conidia and cigar-shaped yeast forms of *S. schenckii s str* (black arrow) were observed in the macrophages. However, the percentage of phagocytosing cells and the mean number of ingested fungi in macrophages were lower in the untreated group (**A**) than in the heteroglycan-treated group (**B**). Scale bar, 50 μm. The phagocytic index of macrophages was higher in the heteroglycan-treated group than in the untreated group (**C**). ***, *p* < 0.001. The data of phagocytosis assay were performed in triplicate

Conidial phagocytosis by macrophages from uninfected mice was observed after incubation for 48 h (Fig. 4A, B) since we wanted to observe the survival of fungus in the macrophages. The phagocytic index (PI) was higher in the heteroglycan-treated group (7.40 ± 1.09) than in the control group (2.68 ± 0.74) (*p* < 0.001), which suggested that heteroglycan promoted the ingestion of conidia by macrophages (Fig. 4C). In addition, ingested conidia could survive and transform to the yeast phase inside the macrophages (Fig. 4A, B). This finding indicated that macrophages played limited roles in the fungicidal ability.

### Cytokine induction in vitro

**Fig. 5:**
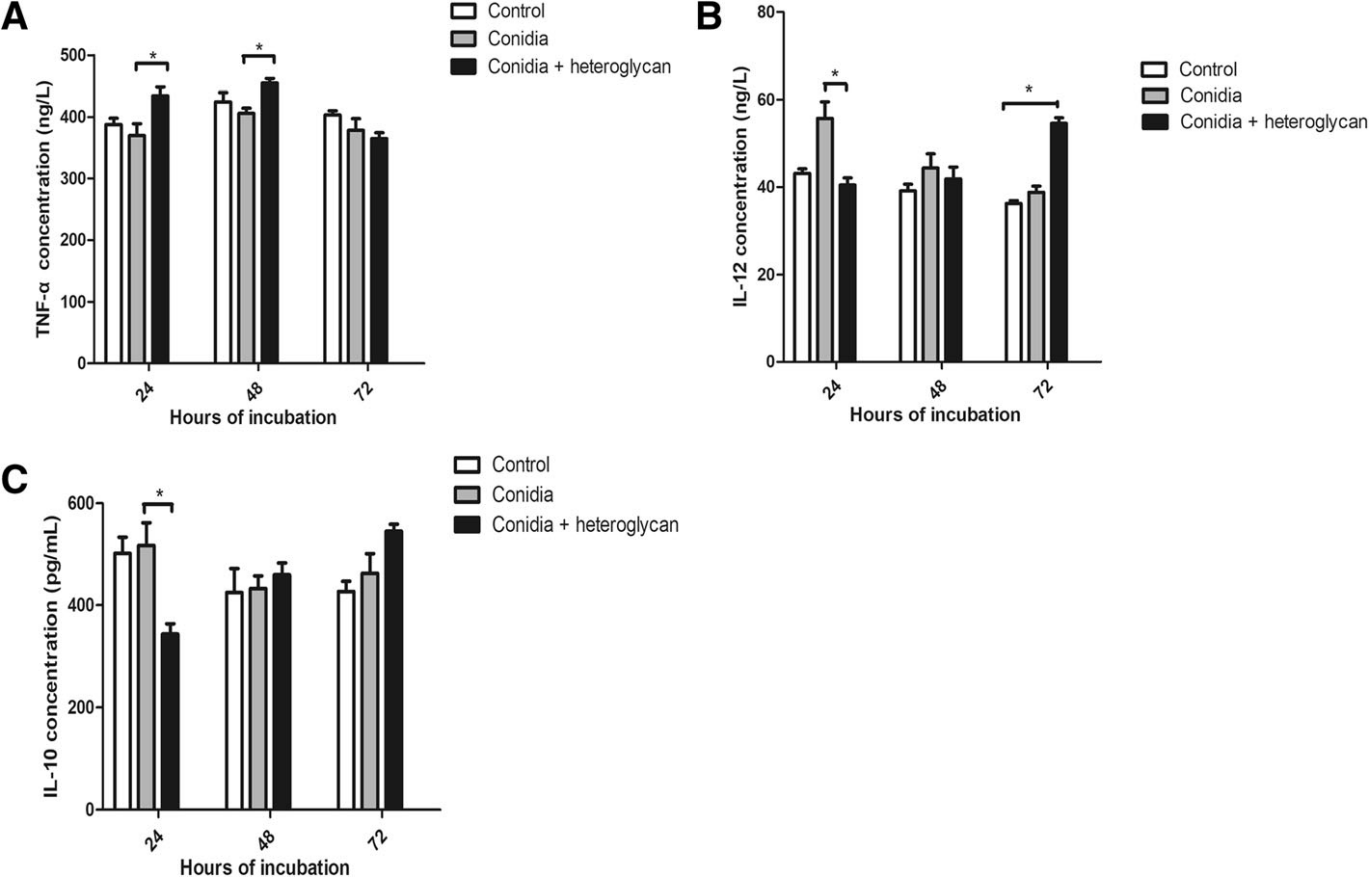
Heteroglycan stimulated cytokine secretion at different time points. **A** TNF-α expression was significantly upregulated at 24 h and 48 h upon heteroglycan treatment compared to that of the untreated cells. *, *p* < 0.05. **B** IL-12 expression was significantly upregulated with conidial stimulation at 24 h compared to that after heteroglycan stimulation. *, *p* < 0.05. However, IL-12 expression gradually increased within 72 h with heteroglycan treatment compared to the control. **C** IL-10 expression increased significantly with conidial stimulation at 24 h compared to heteroglycan stimulation. *, *p* < 0.05. The data were from a representative experiment performed in triplicate

To evaluate the impact of heteroglycan on the pattern of cytokines in the incubation supernatant of macrophages and conidia, we next performed ELISAs of TNF-α, IL-12, and IL-10. The results showed that TNF-α expression was significantly upregulated at 24 h and maintained at a high level at 48 h upon heteroglycan treatment compared to that of the untreated group (*p* = 0.048, *p* = 0.037, respectively) (Fig. 5A). IL-12 expression was significantly upregulated at 24 h but subsequently reduced with conidia stimulation compared to heteroglycan treatment (*p* = 0.018). However, the IL-12 levels gradually increased within 72 h upon heteroglycan treatment compared to the control levels (*p* = 0.013) (Fig. 5B). IL-10 expression was significantly upregulated with conidial stimulation at 24 h and was reduced upon heteroglycan treatment (*p* = 0.048) (Fig. 5C). The upregulation of TNF-α and IL-12 expression and the reduction in IL-10 expression upon heteroglycan treatment might favor the antifungal response since macrophages alone could not exert fungicidal effects.

## Discussion

The fungal cell wall is composed of many polysaccharides that can be recognized by immune cells, regulate cytokine secretion, and modulate immune responses [16, 17]. The culture media, growth conditions and drug treatment may impact fungal morphologies and cell wall composition [11, 15, 18]. There are three morphologies in *S. schenckii s str*: conidia, germlings and yeast-like cells [9]. In the present study, the immune activity of chitin-rich heteroglycan extracted from *S. schenckii s str* were investigated.

Chitin-rich heteroglycan was extracted from the mycelial form of *S. schenckii s str* since it was difficult to collect enough conidia to extract polysaccharides required in our study. The heteroglycan included chitin, mannan, and glucan, which indicated that all these polysaccharides might affect immune activity. However, chitin accounted for 89% of heteroglycan, indicating a greater chance of interacting with immune cells. The particle size of the heteroglycan was determined by flow cytometry since the immune activity of chitin and glucan might be affected by different sizes [19, 20]. In previous studies, chitin was reported to stimulate various immune responses, including pro- and anti-inflammatory immune responses and “allergic” responses, which might be related to differences in size [4, 21–23]. *Da SC* et al. reported that intermediate-sized chitin (40–70 μm) and small chitin (< 40 μm, largely 2–10 μm) stimulated TNF-α secretion, while only small chitin induced IL-10 secretion. Large chitin fragments were nonimmunogenic [19]. Approximately 99.5% of heteroglycan was less than 10 μm in our study, indicating that it could be easily internalized by macrophages and thus modulate immune response. Therefore, we further investigated the immunomodulatory effect of heteroglycans in vivo and in vitro.

Heteroglycans were commonly administered via intraperitoneal inoculation or intranasal instillation in previous studies [4, 7, 24]. The heteroglycan was administered via intraperitoneal inoculation in the mouse model of sporotrichosis in our study. Our data showed that the skin lesions regressed and granulomatous inflammation was reduced with less inflammatory cell infiltration, tissue impairment and fungal burden in the heteroglycan-treated group than in the untreated group. The results above suggested that heteroglycan might play protective roles in mouse defense against fungal infection. These findings were consistent with the results of a previous study showing that chitin elevated the survival of murine candidiasis [5]. However, chitin coupled with glucan from *Aspergillus niger* was associated with eosinophilic allergic inflammation in the lungs of mice, which was reduced following fungal challenge [23]. In fact, polysaccharides derived from different fungi might result in various immune responses that influence the outcome or state of diseases. Therefore, the immune activity of polysaccharides needs to be specifically investigated in species.

To explore the underlying mechanisms for the protective immunity of chitin-rich heteroglycan, we further investigated fungal phagocytosis and cytokine secretion by macrophages upon heteroglycan treatment. One study reported that mannan, glucan, and glucosamine inhibited fungal phagocytosis by phagocytes since they blocked the receptor for recognition on the surface of phagocytes [25]. However, we found that heteroglycan could promote fungal ingestion by macrophages in the present study. This finding might be related to the size of heteroglycans since different sizes and lengths of chitin could bind intracellular or extracellular pattern recognition receptors (PRRs) and thus induce pro- or anti-inflammatory cytokines, which might impact the phagocytosis function of macrophages [4, 21, 22]. *Goodridge* et al. also reported that particulate glucans but not soluble glucans could activate the dectin-1 signaling pathway [20]. In addition, the heteroglycan used in this study was composed of chitin, mannan, and glucan. These polysaccharides could be recognized by PRRs and thus activated macrophages, including increased PRR expression and chitinase secretion, which might promote fungal phagocytosis in previous studies [16]. For example, *Barbara* et al. reported that chitin upregulated TLR2 and TLR4 transcription in keratinocytes [26]. Chitin linked covalently to glucan from *A. fumigatus* induced TNF-α production compared with individual polysaccharides [7]. The alkali-insoluble glucans from *S. schenckii s str* increased NO production by peritoneal macrophages from infected mice [13]. *Goncalves* et al. also reported that alkali-insoluble glucans from *S. schenckii s str* stimulated proinflammatory cytokine IL-1β, IL-18 and IL-17 secretion by coculture with splenocytes and peritoneal macrophages [14]. Furthermore, the upregulated PRR expression of activated macrophages showed a cooperative and synergistic effect in pathogen-associated molecular patterns (PAMPs) recognition. One study reported higher cytokine production in macrophages upon the synergistic response from dectin-1 recognition of glucan and TLR-4 recognition of mannan [27]. These results indicate that innate immunity can be “trained” to acquire a higher capacity to defend against invasive infections, which makes sense to evolution [28, 29]. Indeed, we observed that more conidia were ingested by a single macrophage upon heteroglycan treatment. After incubation for 48 h, the ingested conidia survived and transformed to the yeast phase in macrophages, indicating the limited fungicidal ability of macrophages in vitro. We selected the 48 h time point due to the slow transformation and propagation of *S. schenckii s str* based on our experience. We further investigated the cytokines produced by macrophages with or without heteroglycan treatment. Usually, Th1/Th17 immune responses are considered protective, whereas Th2 immune responses might induce the dissemination of fungi [30]. Cytokines play critical roles in the immune responses of macrophages and the subsequent adaptive response. TNF-α expression was significantly upregulated early upon heteroglycan treatment, suggesting that macrophages were activated and proinflammation might favor fungal clearance. IL-12 expression was temporally upregulated at 24 h with conidia stimulation, which might be insufficient to maintain the efficiency of the adaptive immune response. However, IL-12 expression following co-stimulation of heteroglycan and conidia gradually increased within 72 h, which might have sufficient time for the activation of immune cells. The upregulation of anti-inflammatory IL-10 expression by conidia stimulation at 24 h, which was consistent with the timeframe of IL-12 elevation, might be detrimental to fungal clearance. *Wagener* et al. reported that chitin could stimulate IL-10 secretion by macrophages, which dampened inflammation and maintained immune homeostasis after the pathogen was defeated [4]. However, the IL-10 level was reduced upon heteroglycan treatment which might partly result from the survival of fungi within macrophages in our study. Chitin-rich heteroglycan increased pro-inflammatory cytokine secretion and reduced anti-inflammatory cytokine secretion at different time points in our study, which might involve macrophage polarization and thus modulate the adaptive immune response.

This study had three main limitations. First, the type of macrophages activated by chitin-rich heteroglycan was not investigated. Second, the heteroglycan extracted from *S. schenckii s str* included chitin, mannan, and glucan, and therefore, we could not determine which component was the most important. Third, the heteroglycan used in our study was extracted from the mycelial form of *S. schenckii s str*; thus, the role of polysaccharides from the yeast form needs to be investigated in the future.

## Conclusions

In this study, we investigated the immunomodulation of chitin-rich heteroglycan extracted from *S. schenckii s str* in vivo and in vitro. Chitin-rich heteroglycan potentiated fungal clearance, and the lesions regressed in a mouse model of sporotrichosis. Moreover, chitin-rich heteroglycan promoted fungal phagocytosis by macrophages and elaborately modulated cytokine secretion, which might enhance protection against *S. schenckii s str* infection. Overall, this study demonstrates the immune modulation of chitin-rich heteroglycan which might extend our knowledge about the immune activity of heteroglycan from *S. schenckii s str*.

## Methods

### Animals

Female BALB/c mice, weighing between 20 and 25 g, were purchased from the Animal Center of Sun Yat-sen University. During the experimental period, five or six mice were placed in each mouse cage. These mice were housed under stable conditions (temperature 23–25 °C, relative humidity 50–70%, 12 h light/dark cycle) with unrestricted access to water and diet at the Experimental Animal Center of Sun Yat-sen University. The animal experiments were implemented according to the Guide for the Care and Use of Laboratory Animals of Sun Yat-sen University (Permit Numbers: SCXK 2009–0011) and approved by the Ethics Committee on Animal Experiment in the Faculty of Sun Yat-sen University.

### Microorganisms and inoculum preparation

*S. schenckii s str CBS359.36* (CBS, Utrecht, Netherlands) was used in this study. The fungus was cultured at 25 °C in sabouraud agar medium (SDA) for 7 days to activate the strain and was then inoculated in potato dextrose agar medium (PDA) for an additional 10–14 days to produce conidia. Conidial suspensions were acquired by gently washing the colonies with 5 ml of sterile 1× phosphate buffered saline (1× PBS) (Gibco, USA). Collected conidia were filtered using sterile gauze and then adjusted to the designed concentration in sterile 1× PBS using a hemocytometer. The conidial suspensions were stored at 4 °C and used within 3 days after preparation. Conidial suspension viability was confirmed by culture for 7 days at 25 °C on SDA. Conidia and yeast-like cells are frequent morphologies used for infection in vivo and in vitro. Conidia were used as an infection source in our study since conidia could provide the opportunity to observe the morphological transition within macrophages.

### Heteroglycan extract from *S. schenckii s str*

Heteroglycan was isolated from the mycelial form of *S. schenckii s str* according to previous studies [4, 21] with some adaptations. Briefly, conidia were inoculated on SDA medium at 25 °C for 10–14 days. The fungus was harvested carefully without the medium, washed three times with deionized water, ground to debris, resuspended in 5% (w/v) NaOH and boiled at 100 °C for 5–6 h. The samples were resuspended in 30% hydrogen peroxide/glacial acetic acid solution (1:1) and boiled at 100 °C for 2–3 h. Finally, samples were collected by centrifugation (5000 rpm) and washed three times with deionized water before resuspension in sterile 1× PBS and stored in a 4 °C refrigerator. The samples were hydrolyzed with 13 M (99% (w/v)) trifluoroacetic acid (TFAA) (Jianyang Biotech, Guangdong, China) at 100 °C for 4 h and evaporated at 70 °C. The samples were washed twice with deionized water by evaporation and then resuspended in deionized water. To investigate the components of the heteroglycan, samples were analyzed together with carbohydrate standards by high-performance liquid chromatography according to a peak area normalization method in a carbohydrate analyzer system (Aglient 1100, USA). Before the infection experiments, heteroglycan samples were tested for endotoxin contamination and protein using the limulus amebocyte lysate (LAL) assay (QCL-1000; Lonza) and BCA Protein Assay Kit (CWbio, China) respectively. The heteroglycan samples were inoculated on SDA for 96 h or LB broth for 24 h to detect possible fungal and bacterial contamination. The heteroglycan size was determined by flow cytometry using 2 μm and 10 μm latex beads as size standards (SPH-ACBP, USA), as described in previous studies [4].

### Heteroglycan stimulation in a mouse model of sporotrichosis

Chitin-rich heteroglycan (100 μg/ml) and conidial suspensions of 1 × 10^7^ cfu/ml in sterile 1 × PBS were prepared. Thirty BALB/c mice were randomly assigned into three groups (*n* = 30). Ten mice were subcutaneously injected with 0.10 ml of PBS on the back skin as the blank group (*n* = 10). Another ten mice were subcutaneously injected with 0.10 ml of conidial suspension on the back skin and intraperitoneally injected with 1× PBS as the untreated group (n = 10). The remaining ten mice were subcutaneously injected with 0.10 ml conidial suspension on the back skin and intraperitoneally inoculated with heteroglycan (100 μg) as the heteroglycan-treated group (n = 10). All mice were observed for 5 weeks, and the lesion sizes were measured weekly. At the end of the experiment, the mice were sacrificed by cervical dislocation. The skin lesions, lung, liver, and spleen were harvested under aseptic conditions for fungal culture. The pathologic differences and fungal burden in local skin were evaluated by H&E and PAS staining.

### Macrophages isolation from mice

Peritoneal macrophages of 36 healthy mice that were sacrificed by cervical dislocation were collected from the peritoneal cavity lavage using sterile 1× PBS. More than 95% of the collected cells were macrophages, as demonstrated by Giemsa staining. Macrophages were adjusted to 1 × 10^6^ cells/well in 6-well flat-bottom culture plates (Corning, Beijing). The plates with cells were incubated for 2 h at 37 °C in an incubator containing 5% CO_2_ and 95% air. After incubation, the wells were washed with DMEM (low glucose) (Gibco, USA) 3 times to remove nonadherent cells, and the adherent cells were cultured in DMEM (low glucose) containing 10% fetal bovine serum/newborn calf serum (Gibco, USA) for the following phagocytosis and cytokine induction assay.

### Phagocytosis assay

Conidia (5 × 10^6^ cfu/well) with or without chitin-rich heteroglycan (10 μg/ml) were incubated with macrophages (1 × 10^6^ cells/well) in round-bottom 6-well plates with slides at the bottom (MOI 5:1). All plates were incubated at 37 °C in 5% CO_2_ and 95% air for 48 h. The supernatant was removed and washed with 1× PBS 3 times. The slides were stained with eosin (Sigma, USA) and analyzed under an optical microscope (400× magnification; Axiostar plus). The phagocytic index (PI) was calculated as the percentage of phagocytosing cells multiplied by the mean number of ingested fungi in a total of 200 macrophages per well [31].

### Cytokine induction assay

Conidial suspensions (1 × 10^6^ cfu/well) were incubated with macrophages (1 × 10^6^ cells/well) in 6-well plates. Chitin-rich heteroglycan (10 μg/ml) was added to 4 wells as the heteroglycan-treated group while the same volume of DMEM (low glucose) was added to another 4 wells as the conidia group. The remaining 4 wells contained macrophages without conidia or heteroglycan as the control group. All plates were incubated for 24 h, 48 h, and 72 h at 37 °C in an incubator containing 5% CO_2_ and 95% air. After the appropriate incubation time, supernatants were collected to evaluate TNF-α, IL-12, and IL-10 secretion using commercial enzyme-linked immunosorbent assays (ELISAs) (R&D System, USA) following the manufacturer’s instructions. Colorimetric reactions were measured at 450 nm with wavelength correction at 570 nm on a Multiskan Ascent ELISA reader (Labsystems, Helsinki, Finland).

### Statistical analysis

Statistical analysis was performed using SPSS software 22.0 (SPSS Inc., Chicago, IL, USA). Data represented cumulative results of all experiments and were showed as mean ± standard deviation (SD). The Mann-Whitney U test was used to establish statistical significance of phagocytosis and fungal burden in two groups. Kruskal-Wallis test was used to establish statistical significance of cytokines secretion in three groups. A value of *p* < 0.05 was considered statistically significant.

## Data Availability

All data generated or analyzed during this study are included in this published article. Access to raw data can be acquired by connecting to the corresponding author via email.

## Abbreviations

HPLC: High-performance liquid chromatography
PI: phagocytic index
ELISA: Enzyme-Linked Immunosorbent Assays
CFU: Colony-forming unit
H&E: Hematoxylin and eosin
PAS: Periodic acid-Schiff
PRRs: Pattern recognition receptors
PAMPs: Pathogen-associated molecular patterns
